# Development and validation of a rabbit model of *Pseudomonas aeruginosa* hyperdynamic septic shock for preclinical drug development

**DOI:** 10.1128/aac.01936-25

**Published:** 2026-06-15

**Authors:** Nguyen D. Tran, Nhu T. Q. Nguyen, Trang T. T. Vu, Phung-Hieu Diep, Naomi N. Villescas, Nhi N. Y. Nguyen, Kei M. Sato, William J. Weiss, Thien N. M. Doan, Binh An Diep

**Affiliations:** 1 Division of HIV, Infectious Diseases, and Global Medicine, Department of Medicine, University of California San Francisco 8785, San Francisco, California, USA; 2 University of Medicine and Pharmacy, Ho Chi Minh City, Vietnam; 3 Evergreen Valley College, San Jose, California, USA; 4 University of Science, Viet Nam National University, Ho Chi Minh City, Vietnam; 5 Pre-Clinical Services at UNT Health Science Center, Fort Worth, Texas, USA; Entasis, Big Bay, Michigan, USA

**Keywords:** *Pseudomonas aeruginosa*, septic shock, hyperdynamic state, rabbit model, preclinical efficacy model validation, mechanical ventilation, fluid resuscitation, vasopressor

## Abstract

Translating preclinical findings into clinical success remains a major barrier in the development of antimicrobials targeting *Pseudomonas aeruginosa*, particularly for severe infections such as sepsis and septic shock. Rodent models frequently fail to predict therapeutic efficacy in clinical sepsis. To address this gap, we developed and validated a rabbit model of hyperdynamic septic shock incorporating intensive care unit (ICU)-style monitoring and supportive care to better reflect human clinical practice. Rabbits challenged intravenously with *P. aeruginosa* progressed predictably from hyperdynamic circulation to hypodynamic shock, characterized by progressive metabolic acidosis, leukocyte dysregulation, and acute myocardial injury. A sustained 20% decline in mean arterial pressure reliably marked the onset of cardiovascular decompensation and was prospectively applied in follow-on studies as a predefined hemodynamic trigger for initiating therapeutic interventions. Despite pharmacokinetic studies showing that ciprofloxacin 30 mg/kg infused over 1 h every 8 h achieved human-equivalent exposures and exceeded the AUC_0–24h_/MIC ≥ 125 efficacy threshold, ciprofloxacin monotherapy improved survival compared to placebo but failed to prevent progression to fatal shock. In contrast, the combination of ciprofloxacin and protocolized supportive care—based on the clinical “VIP” strategy (ventilation, infusion, and pressor)—rescued 75% of animals from lethal septic shock compared with VIP plus placebo, while also stabilizing physiological parameters and markedly reducing bacterial burdens. Together, these findings underscore the importance of VIP supportive care for maintaining hemodynamic stability and establish a clinically relevant platform for evaluating antimicrobial therapies.

## INTRODUCTION

Evaluating new drugs targeting antimicrobial-resistant pathogens, such as *Pseudomonas aeruginosa*, in clinical trials has proven particularly challenging for severe sepsis and septic shock, which are associated with high mortality rates (1). Mouse sepsis models are commonly used in antimicrobial drug discovery due to their low cost, enabling high-throughput screening of potential therapeutic agents (2, 3). However, therapeutic agents shown to be efficacious in mouse sepsis models have rarely been translated into success in clinical trials (4–7). Additional animal systems are often required to better bridge preclinical findings to human trials. Non-human primates, particularly rhesus macaques, have historically been used for this purpose. However, like mice, these Old World monkeys display relative resistance to certain Gram-negative infections, including those caused by *P. aeruginosa* (8), which may be attributed to inherent resistance to lipopolysaccharide (LPS) (9). In contrast, rabbits exhibit susceptibility to LPS that more closely resembles that of humans and chimpanzees (10, 11) and are naturally vulnerable to *P. aeruginosa* infection (12).

To develop a preclinical sepsis model that more accurately predicts human clinical responses and facilitates regulatory approval, we established an experimental intensive care unit (ICU) to conduct pathophysiological and therapeutic efficacy studies that closely replicate clinical practice (13, 14). Using this setup, we demonstrated that rabbits can be effectively instrumented for low-tidal volume mechanical ventilation, advanced hemodynamic monitoring, and serial blood biomarker analyses—tools essential for managing patients with severe sepsis and septic shock (15). Importantly, we showed that rabbits infected with *Staphylococcus aureus*, but not those challenged with saline, exhibited a hyperdynamic phase of septic shock characterized by increased mean arterial pressure (MAP), elevated cardiac output, increased stroke volume, and decreased systemic vascular resistance (15). This was followed by a fatal hypodynamic phase characterized by a rapid drop in MAP, increased central venous pressure, reduced cardiac output and stroke volume, elevated systemic vascular resistance, and decreased left ventricular ejection fraction—closely mirroring the defining clinical features of human staphylococcal septic shock (15).

We report here on two complementary studies that together establish a rabbit model of hyperdynamic septic shock induced by *P. aeruginosa*. The first study was a natural history study designed to evaluate how closely the disease progression in this model mirrors the pathophysiology of human sepsis. This was achieved through advanced hemodynamic monitoring and serial assessments of complete blood counts, blood gas measurements, clinical chemistry panels, and biomarkers of organ dysfunction in anesthetized rabbits intravenously challenged with *P. aeruginosa*. The second study built upon these findings to define evidence-based physiological triggers for initiating therapeutic interventions aimed at halting and reversing septic shock progression. These interventions included administration of an antimicrobial agent, ciprofloxacin, and initiation of a protocolized supportive care regimen modeled after the “VIP” strategy—ventilation, infusion, and pressors—commonly used in human intensive care units to stabilize patients with septic shock (16).

## RESULTS

### Reproducing human septic shock features in a rabbit model of hyperdynamic septic shock with *P. aeruginosa*

To determine whether key features of human severe sepsis and septic shock could be replicated in a rabbit model, we intravenously challenged seven anesthetized outbred rabbits with a lethal dose of 3 × 10^9^ CFU *P*. *aeruginosa* strain 6206. All animals were mechanically ventilated using a lung-protective, low-tidal volume strategy and surgically instrumented with multiple arterial and central venous catheters to enable advanced hemodynamic monitoring (13). Intravenous catheters were also used for continuous drug and fluid administration into the ear veins using an electric infusion pump, allowing for individualized fluid resuscitation and real-time titration of norepinephrine on a minute-to-minute basis as previously described (13).

**Fig 1:**
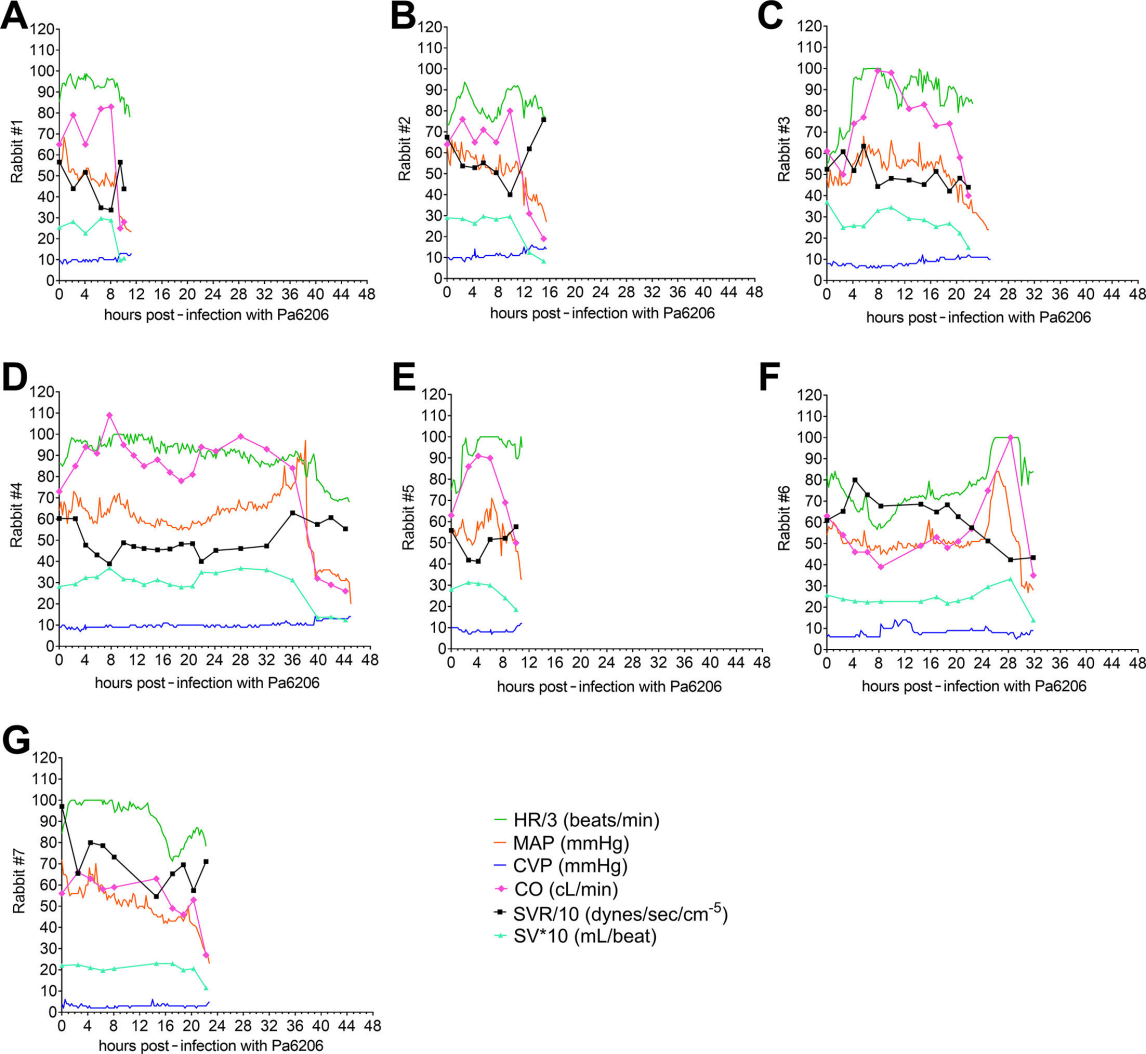
Natural history of hemodynamics during *P. aeruginosa* septic shock in mechanically ventilated rabbits. Seven anesthetized New Zealand White rabbits (**A–G**) were challenged intravenously at 0 h with 3 × 10⁹ CFU of *P. aeruginosa* strain 6206 and monitored continuously without therapy. Each panel shows the time course for a single animal of heart rate (HR), mean arterial pressure (MAP), central venous pressure (CVP), cardiac output (CO), systemic vascular resistance (SVR), and stroke volume (SV). To permit display on a common y-axis, HR is plotted as HR/3, SVR as SVR/10, and SV as SV × 10 (units indicated in the legend). CO was measured serially by thermodilution. MAP and CVP were recorded invasively via arterial and central venous catheters, while animals were maintained on lung-protective mechanical ventilation. Traces end at the humane/terminal endpoint (TE) for each rabbit.

Advanced hemodynamic monitoring revealed a biphasic cardiovascular response in the seven *P. aeruginosa*-infected rabbits (Fig. 1 and Fig. S1). Within 2 to 4 h post-infection, animals entered a hyperdynamic state, characterized by elevated cardiac output and preserved perfusion (15). This was followed by a transition to hypodynamic circulation, marked by progressive declines in both cardiac output and mean arterial pressure (MAP), typically occurring within 2 to 4 h preceding death. Central venous pressure (CVP) rose consistently during the final 2-hour window, temporally aligned with terminal hypoperfusion and cardiovascular collapse (Fig. 1 and Fig. S1).

The median survival time for the seven *P. aeruginosa*–infected rabbits was 16.5 h post-infection, with all animals succumbing to infection by 48 h (Fig. S2A). Quantification of bacterial burdens at necropsy demonstrated disseminated infection across all major organs, with median log₁₀ CFU values ranging from approximately 5.3 in the spleen to 7.9 in the liver (Fig. S2B). These findings confirm systemic bacterial spread and multiorgan involvement.

**Fig 2:**
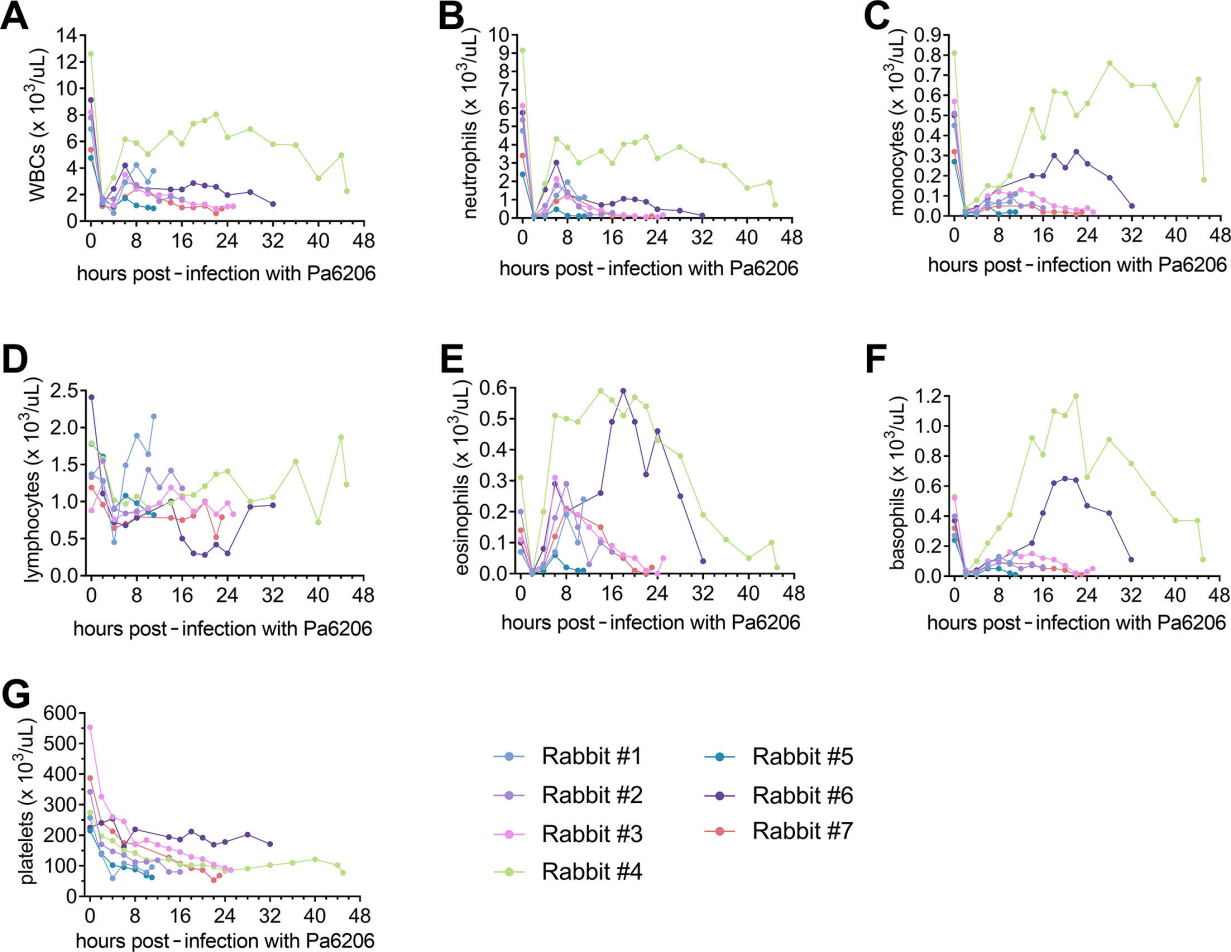
Hematologic responses during *P. aeruginosa* septic shock in the rabbit natural history study. Serial complete blood counts were obtained from arterial samples to quantify changes in (**A**) total white blood cells (WBCs), (**B**) neutrophils, (**C**) monocytes, (**D**) lymphocytes, (**E**) eosinophils, (**F**) basophils, and (**G**) platelets over time. Data shown here were obtained from the same seven rabbits depicted in Fig. 1; refer to the included legend for rabbit identification numbers and corresponding colored traces.

Longitudinal hematologic analyses from the same seven rabbits shown in Fig. 1 revealed rapid-onset cytopenias following intravenous *P. aeruginosa* infection (Fig. 2). Total white blood cell counts declined sharply within 2–4 h post-infection (Fig. 2A), driven primarily by marked neutropenia and monocytopenia (Fig. 2B and C). Platelet counts also decreased progressively over time, indicating developing thrombocytopenia (Fig. 2G). In contrast, eosinophil and basophil counts exhibited transient elevations during the early hyperdynamic phase before declining during the transition to hypodynamic shock (Fig. 2E and F). Collectively, these findings demonstrate acute cytopenias and transient dysregulation of leukocyte subsets characteristic of severe Gram-negative septic shock (17, 18).

**Fig 3:**
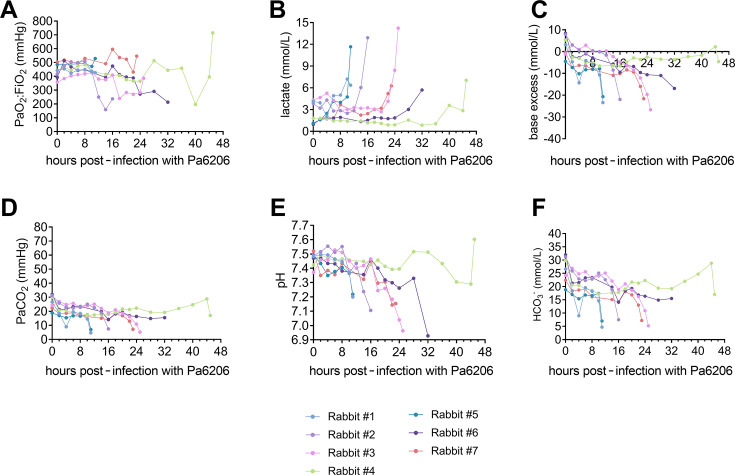
Arterial blood gas and metabolic changes during *P. aeruginosa* septic shock in the rabbit natural history study. Serial arterial blood gases were measured in the same seven mechanically ventilated rabbits shown in Figs. 1 and 2 following intravenous challenge with *P. aeruginosa* strain 6206. Parameters shown include (**A**) arterial oxygenation (PaO₂:FiO₂ ratio), (**B**) lactate, (**C**) base excess, (**D**) arterial carbon dioxide (PaCO₂), (**E**) pH, and (**F**) bicarbonate (HCO₃⁻) over time. Data shown here were obtained from the same seven rabbits depicted in Fig. 1; refer to the included legend for rabbit identification numbers and the corresponding colored traces.

Serial arterial blood gas measurements demonstrated progressive metabolic acidosis and hypoxemia (Fig. 3). Initially, arterial oxygenation (PaO₂:FiO₂) ratios remained stable, indicating preserved gas exchange during the hyperdynamic phase (Fig. 3A). As shock progressed, animals developed rising lactate concentrations, increasingly negative base excess, and declining bicarbonate (HCO₃⁻) levels, reflecting worsening metabolic acidosis (Fig. 3B, C and F). Blood pH values fell steadily over time, reaching markedly acidotic levels preceding cardiovascular collapse (Fig. 3E), while PaCO₂ fluctuated within a range consistent with partial respiratory compensation (Fig. 3D). These coordinated metabolic and gas exchange changes paralleled hemodynamic deterioration, providing physiological evidence of tissue hypoperfusion during *P. aeruginosa*-induced septic shock.

Biomarkers of acute organ injury were measured longitudinally to assess the progression of multiorgan dysfunction during *P. aeruginosa*–induced septic shock (Fig. S3). Acute myocardial injury biomarkers, including cardiac troponin I (cTnI), creatine kinase–MB (CK-MB), and myoglobin, showed sharp increases beginning at the time of a 20% decline in mean arterial pressure (MAP↓20%) and peaking at the terminal endpoint, consistent with acute cardiac injury and circulatory collapse (Fig. S3A through C). Acute liver injury biomarkers, including aspartate aminotransferase (AST), alanine aminotransferase (ALT), the AST/ALT ratio, and total bilirubin levels, were elevated above baseline in only a few animals, indicating that hepatic involvement was limited and variable (Fig. S3D through G). Similarly, acute kidney injury biomarkers, including blood urea nitrogen (BUN) and creatinine levels, remained near baseline in most animals, suggesting limited renal involvement (Fig. S3H through I). Taken together with the hematologic and metabolic derangements described in Fig. 1 through 3, these findings illustrate a coordinated transition from a compensated hyperdynamic state to a terminal hypodynamic phase, culminating in myocardial failure and death in the rabbit model of *P. aeruginosa*-induced septic shock.

### MAP decline as a surrogate trigger for therapeutic intervention

Although cardiac output is a critical parameter for tracking septic shock progression, its measurement via thermodilution is invasive and carries procedural risks (Fig. 1). In contrast, we observed that the time when MAP declined by 20% from baseline (denoted as T_MAP↓20%_) occurred at a median of 15.8 h post-infection (IQR: 10.5–29.9 h), marking the onset of hypodynamic circulation. This decline preceded the terminal endpoint (median 22.8 h; IQR: 11.2–31.9 h) by approximately 4.6 h (IQR: 2.0–6.7 h) (Fig. 1), potentially providing a reproducible and clinically actionable window for therapeutic intervention. The consistency of T_MAP↓20%_ and its temporal proximity to terminal decompensation suggest that it is a reliable, minimally invasive surrogate marker and clinically relevant trigger for intervention in the rabbit model of hyperdynamic septic shock with *P. aeruginosa*.

### Establishing a clinically relevant ciprofloxacin dosing regimen

**Fig 4:**
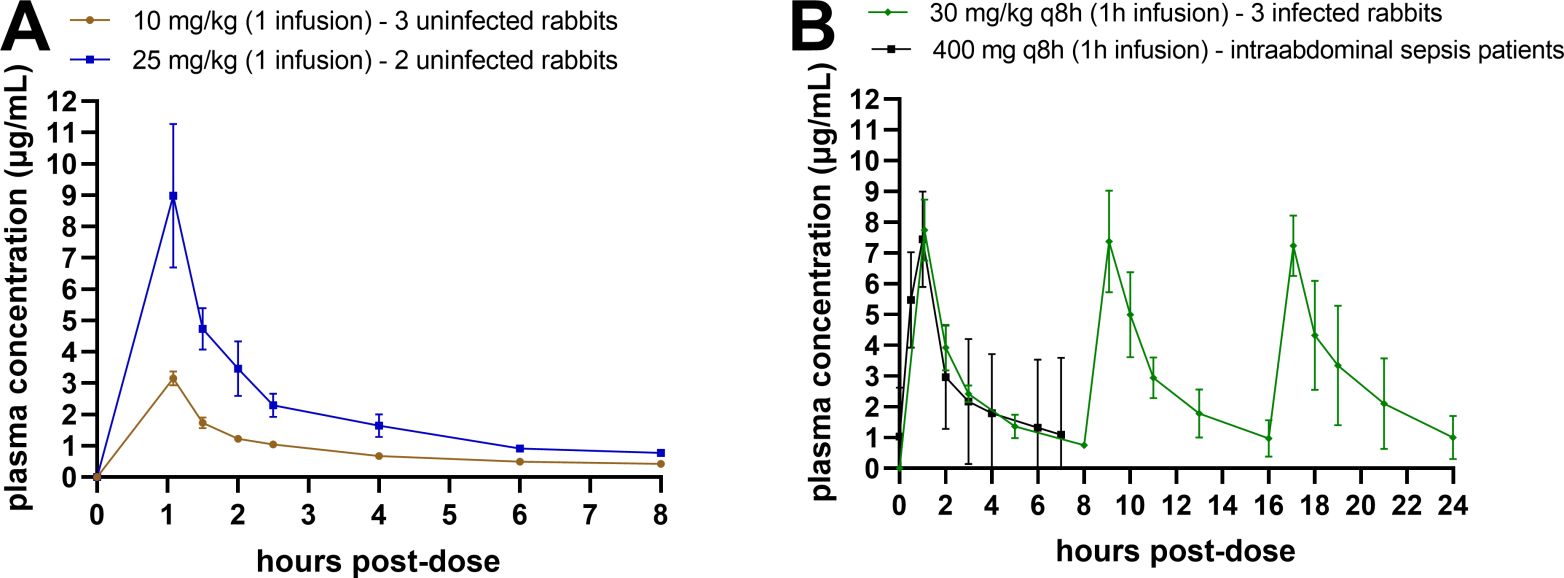
Ciprofloxacin plasma concentration–time profiles in rabbits and comparison to human data. (**A**) Plasma ciprofloxacin concentrations in uninfected rabbits following a single 1-hour intravenous infusion of 10 mg/kg (brown line, *n* = 3) or 25 mg/kg (blue line, *n* = 2). (**B**) Plasma ciprofloxacin concentrations in infected rabbits administered ciprofloxacin at 30 mg/kg every 8 h by 1-hour infusion (green line, *n* = 3), compared to published data from adult patients with intra-abdominal sepsis receiving 400 mg every 8 h by 1-hour infusion (black line; data from reference 20). Data are presented as mean ± standard deviation.

To support the translational relevance of the rabbit model, we sought to identify a human-equivalent ciprofloxacin dosing regimen that achieves pharmacokinetic/pharmacodynamic (PK/PD) exposures predictive of clinical efficacy. As an initial step, total plasma ciprofloxacin concentrations (bound and unbound) were measured in uninfected rabbits following a single 1-hour intravenous infusion of 10 mg/kg or 25 mg/kg. The 10 mg/kg dose yielded a mean 24-hour area under the concentration-time curve to minimum inhibitory concentration ratio (AUC_0–24h_/MIC, MIC = 0.5 µg/mL for Pa6206) of 45.6 (95% CI, 43.5–47.6), whereas the 25 mg/kg dose resulted in a mean AUC_0–24h_/MIC of 113.1 (95% CI, 95.3–130.9) (Fig. 4A). Given that the established pharmacodynamic efficacy threshold for ciprofloxacin based on total drug exposure is AUC_0–24h_/MIC ≥125 (19), these findings suggested that a greater dose of 30 mg/kg administered every 8 h (q8h) would be required to achieve clinically relevant exposures in the rabbit model. Indeed, we found that ciprofloxacin PK in infected rabbits administered 30 mg/kg q8h via 1-hour infusion for three doses achieved a mean AUC_0–24h_/MIC of 132.6 (95% CI, 114.2–150.9), exceeding the pharmacodynamic target for Gram-negative pathogens (Fig. 4B). Notably, the plasma concentration–time profiles in rabbits closely mirrored those observed in adult patients with intra-abdominal sepsis treated with 400 mg ciprofloxacin every 8 h by 1-hour infusion, despite interspecies differences in weight-based dosing (20). These data support 30 mg/kg q8h via 1-hour infusion as a human-equivalent regimen and support its use in the following therapeutic efficacy studies in this model.

### Ciprofloxacin monotherapy insufficient to halt lethal septic shock

**Fig 5:**
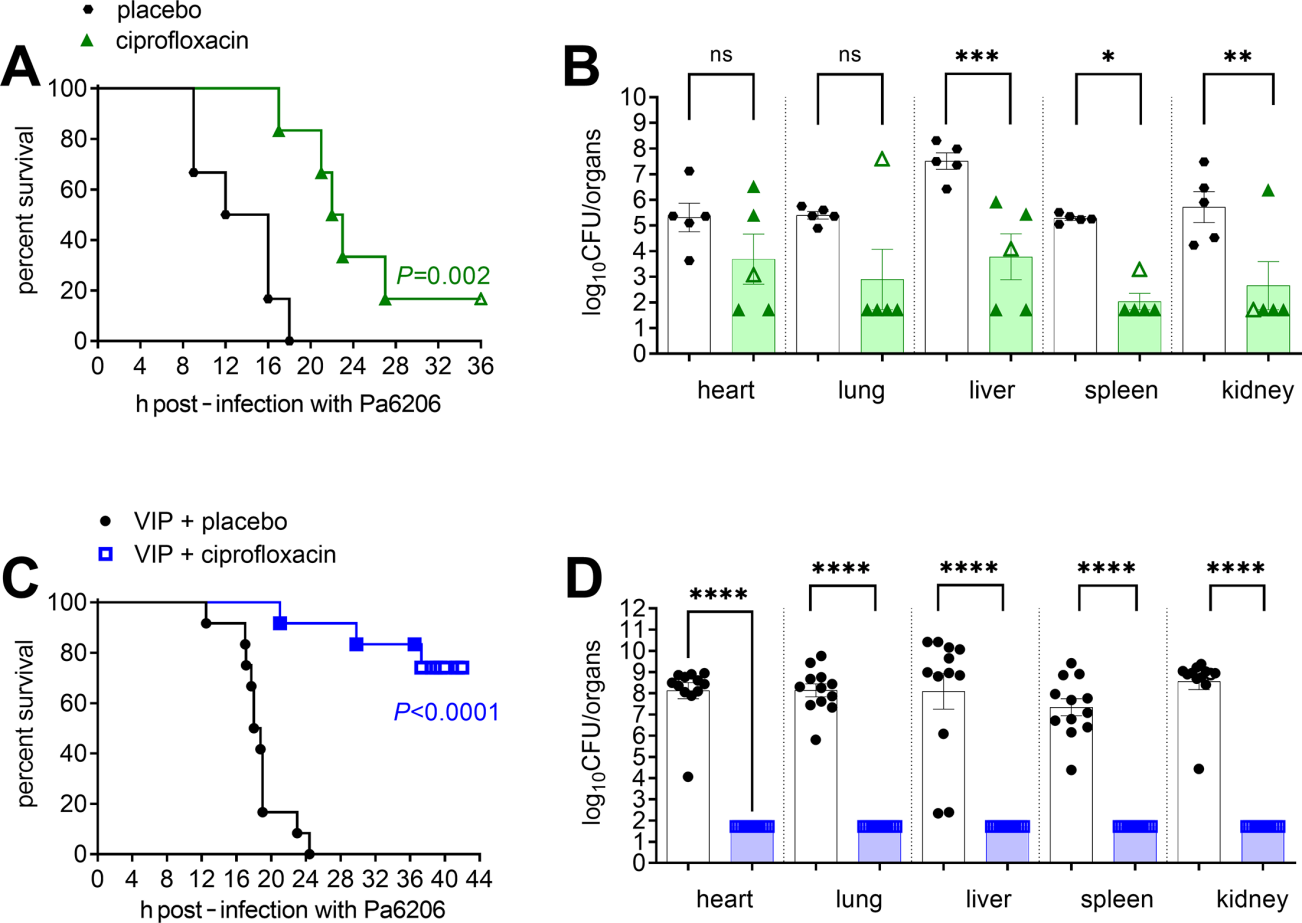
Efficacy of ciprofloxacin with or without ICU-style supportive care (VIP) in a rabbit model of *P. aeruginosa* hyperdynamic septic shock**.** (**A**) Kaplan–Meier survival curves comparing placebo-treated and ciprofloxacin-treated rabbits (30 mg/kg q8h × 3 doses via 1-hour infusion; *n* = 6). (**B**) Bacterial burdens in major organs from the same rabbits showing reduced CFU counts in ciprofloxacin-treated animals, consistent with antimicrobial activity. (**C**) Survival curves for rabbits receiving ICU-style “VIP” supportive care (mechanical ventilation, fluid infusion, and vasopressor support) plus placebo (*n* = 12) or combined VIP + ciprofloxacin therapy (30 mg/kg q8h × 3 doses; *n* = 12). (**D**) Quantification of bacterial burdens in major organs. Each symbol represents an individual animal; bars indicate median and IQR. ns, not significant; *P < 0.05; **P < 0.01; ***P < 0.001; ****P < 0.0001.

To evaluate whether a human-equivalent dosing regimen of ciprofloxacin could prevent lethal septic shock, we conducted a pilot efficacy study in which 12 rabbits infected with *P. aeruginosa* were randomized at T_MAP↓20%_ to receive either ciprofloxacin (30 mg/kg q8h × 3 doses, administered over 1 h) or placebo (normal saline infused at an equivalent rate). Treatment was initiated at T_MAP↓20%_, corresponding to a median [IQR] of 8.0 h [6.3–8.7] post-infection for placebo and 9.5 h [6–13] post-infection for ciprofloxacin (*P* = 0.33, Mann–Whitney U test). Ciprofloxacin-treated rabbits demonstrated significantly prolonged survival compared to controls (*P* = 0.002 by log-rank test); however, five of six ciprofloxacin-treated animals and all six saline-treated animals ultimately progressed to lethal septic shock (Fig. 5A). Among the ciprofloxacin-treated rabbits that succumbed, one died after receiving a single dose, three after two doses, and one after completing the full three-dose regimen. The sole survivor in the ciprofloxacin group received all three doses. Bacterial burdens in the saline-treated group were comparable to those observed in the natural history cohort (Fig. 5B), while ciprofloxacin-treated rabbits exhibited lower CFU counts in target organs, consistent with antimicrobial activity. Blood samples were not collected for hematologic or serum chemistry analysis in this pilot study. Collectively, these findings indicate that although ciprofloxacin reduced bacterial burden and modestly extended survival, monotherapy initiated at T_MAP↓20%_ was insufficient to prevent progression to lethal septic shock in most animals.

### Ciprofloxacin and ICU-supportive care halted lethal septic shock

To evaluate whether ICU-style life-sustaining supportive care—based on the clinical “VIP” strategy of ventilation, infusion, and vasopressor support—enhances the efficacy of ciprofloxacin, we randomized 24 *P. aeruginosa*-infected rabbits at T_MAP↓20%_ into two treatment groups: (i) fluid resuscitation with saline and norepinephrine plus ciprofloxacin (30 mg/kg, q8h × 4 doses, administered over 1 h), referred to as VIP plus ciprofloxacin; or (ii) saline and norepinephrine plus placebo (volume-matched saline), referred to as VIP plus placebo. Treatment allocation was not blinded because a visually matched vehicle control for the yellow ciprofloxacin solution in 5% dextrose (Hospira) could not be prepared. Treatment was initiated at T_MAP↓20%_, corresponding to a median [IQR] of 11.7 h [9.1–13.4] post-infection for VIP plus placebo and 9.8 h [7.3–10.6] post-infection for VIP plus ciprofloxacin (*P* = 0.23; Table 1).

**TABLE 1:** Baseline characteristics and outcomes of treatment with VIP plus placebo vs VIP plus ciprofloxacin in a rabbit model of hyperdynamic septic shock with *P. aeruginosa^a^*

	Sampling time	VIP + placebo (12 rabbits)	VIP + ciprofloxacin (12 rabbits)	*P*-values
Mean arterial pressure (mmHg)	Pre-infection	50 (44–53)	54 (50–56)	0.12
	TTx	39 (36–42)	42 (40–45)	0.09
	TTx + 6 h	48 (42–50)	54 (47–61)	0.09
	TE	28 (25–32)	50 (41–57)	**<0.001**
PaO_2_:FiO_2_ (mmHg)	Pre-infection	523 (451–557)	519 (460–550)	0.93
	TTx	521 (494–552)	531 (482–569)	0.93
	TTx + 6 h	493 (334–553)	517 (514–540)	0.92
	TE	324 (224–554)	412 (261–467)	0.92
PaCO_2_ (mmHg)	Pre-infection	35 (32–41)	33 (30–38)	0.77
	TTx	36 (32–45)	33 (29–38)	0.62
	TTx + 6 h	37 (36–47)	38 (29–45)	0.82
	TE	38 (22–44)	40 (31–44)	0.86
pH	Pre-infection	7.50 (7.45–7.54)	7.47 (7.42–7.52)	0.58
	TTx	7.34 (7.28–7.39)	7.38 (7.32–7.46)	0.26
	TTx + 6 h	7.15 (7.01–7.30)	7.34 (7.18–7.37)	0.10
	TE	6.84 (6.68–6.92)	7.30 (7.11–7.40)	**0.003**
Base excess (mmol/L)	Pre-infection	3.3 (1.5, 5.8)	1.5 (−0.7, 3.8)	0.29
	TTx	−5.0 (−7.6, −4.2)	−4.6 (−9.2, −0.4)	0.49
	TTx + 6 h	−15.4 (−19.4, −8.1)	−5.6 (−9.2, −2.2)	0.06
	TE	−26.9 (−30.5, −25.3)	−7.9 (−17.7, −2.7)	**0.002**
Lactate (mmol/L)	Pre-infection	2.6 (1.9–3.9)	3.0 (2.3–5.7)	0.44
	TTx	6.5 (5.0–8.0)	4.0 (3.2–7.8)	0.32
	TTx + 6 h	9.5 (7.7–10.9)	5.5 (4.1–7.6)	0.20
	TE	14.1 (11.4–16.0)	6.3 (2.9–9.8)	**0.005**
Cardiac troponin I (ng/mL)	Pre-infection	0 (0–0.02)	0 (0–0.01)	0.76
	TTx	0.33 (0.04–0.72)	0.09 (0.03–0.43)	0.76
	TTx + 6 h	1.50 (0.15–4.36)	0.46 (0.05–2.00)	0.76
	TE	7.95 (4.68–10.11)	4.80 (1.57–13.69)	0.76
CK-MB (ng/mL)	Pre-infection	3.1 (1.9–4.5)	5.3 (2.1–6.3)	0.17
	TTx	8.1 (5.5–12.8)	5.5 (3.6–15.0)	0.25
	TTx + 6 h	45.0 (21.4–66.0)	12.0 (6.1–31.0)	0.17
	TE	147.3 (107.2–254.5)	15.8 (4.7–30.8)	**0.001**
Myoglobin (ng/mL)	Pre-infection	11 (3–28)	15 (3–22)	0.77
	TTx	106 (73–430)	26 (8–73)	0.040
	TTx + 6 h	1,120 (175–1,720)	108 (8–265)	0.06
	TE	4,575 (2,678–6,900)	66 (4–626)	**<0.001**
ALT (U/L)	Pre-infection	33 (18–51)	32 (20–59)	0.90
	TTx	40 (20–68)	40 (19–59)	0.90
	TTx + 6 h	80 (23–98)	40 (23–65)	0.59
	TE	91 (20–169)	44 (25–73)	0.34
AST (U/L)	Pre-infection	34 (20–71)	31 (23–49)	0.98
	TTx	100 (82–182)	116 (104–150)	0.98
	TTx + 6 h	261 (174–444)	139 (133–149)	**0.020**
	TE	555 (226–930)	92 (54–227)	**0.001**
ALP (U/L)	Pre-infection	118 (89–138)	116 (100–136)	0.99
	TTx	88 (76–114)	89 (79–112)	0.99
	TTx + 6 h	95 (86–123)	75 (67–109)	0.43
	TE	122 (111–141)	65 (54–94)	**0.020**
BUN (mg/dL)	Pre-infection	23 (20–27)	26 (23–32)	0.21
	TTx	36 (34–40)	37 (33–43)	0.85
	TTx + 6 h	35 (30–47)	29 (28–36)	0.34
	TE	37 (33–43)	37 (14–43)	0.85
Creatinine (mg/dL)	Pre-infection	1.1 (0.9–1.9)	1.4 (1.0–1.8)	0.81
	TTx	1.4 (1.2–2.5)	1.7 (1.4–1.8)	0.81
	TTx + 6 h	2.2 (1.3–2.4)	1.3 (0.9–1.5)	0.11
	TE	1.9 (1.3–2.2)	1.8 (0.9–3.1)	0.82
Glucose (mg/dL)	Pre-infection	160 (158–174)	171 (159–192)	0.55
	TTx	121 (89–135)	126 (112–142)	0.87
	TTx + 6 h	110 (58–248)	147 (128–181)	0.87
	TE	120 (63–271)	211 (155–329)	0.14
K^+^ (mmol/L)	Pre-infection	4.1 (3.6–5.0)	4.5 (3.8–4.8)	0.83
	TTx	4.4 (3.6–5.4)	4.1 (3.6–4.3)	0.68
	TTx + 6 h	6.4 (4–7.1)	3.9 (3.6–4.2)	0.11
	TE	8.5 (8.5–8.5)	5.0 (3.4–8.5)	**0.040**
Albumin (g/L)	Pre-infection	28.8 (27.5–32.0)	32.4 (29.3–34.2)	0.35
	TTx	23.4 (21.6–27.5)	24.3 (23.4–28.4)	0.54
	TTx + 6 h	18.0 (18.0–23.4)	21.6 (19.8–25.2)	0.35
	TE	18.0 (18.0–18.0)	18.0 (18.0–18.0)	0.54
White blood cell (× 10^3^/µL)	Pre-infection	6.31 (5.08–7.65)	6.81 (6.06–7.78)	0.73
	TTx	1.78 (1.46–2.38)	1.94 (1.71–3.26)	0.73
	TTx + 6 h	2.76 (2.10–4.05)	2.78 (2.40–4.29)	0.76
	TE	2.8 (1.81–3.95)	8.74 (5.14–10.35)	**<0.001**
Neutrophil (× 10^3^/µL)	Pre-infection	4.14 (3.09–4.99)	4.77 (3.90–5.53)	0.36
	TTx	0.18 (0.15–0.37)	0.55 (0.31–1.49)	**0.040**
	TTx + 6 h	0.22 (0.13–0.51)	0.81 (0.46–1.55)	**0.040**
	TE	0.27 (0.14–0.52)	2.03 (1.04–5.52)	**<0.001**
Monocyte (× 10^3^/µL)	Pre-infection	0.41 (0.31–0.50)	0.51 (0.45–0.57)	0.14
	TTx	0.04 (0.03–0.07)	0.06 (0.03–0.13)	0.45
	TTx + 6 h	0.05 (0.04–0.06)	0.14 (0.08–0.17)	**0.002**
	TE	0.05 (0.03–0.11)	0.74 (0.35–1.37)	**<0.001**
Platelet (× 10^3^/µL)	Pre-infection	300 (265–376)	407 (356–421)	0.09
	TTx	127 (95–148)	149 (115–203)	0.21
	TTx + 6 h	88 (53–123)	129 (94–202)	0.07
	TE	68 (52–86)	136 (86–203)	**0.005**

^*a*^ *P*-values were calculated using the Mann–Whitney U test with Holm–Šidák correction for multiple comparisons between experimental groups (VIP + placebo vs VIP + ciprofloxacin) at four independent time points: pre-infection, time of treatment (TTx), 6 h after time of treatment (TTx + 6 h), and terminal endpoint (TE). *P*-values < 0.05 are shown in bold.

Protocolized VIP supportive care was implemented as follows. At T_MAP↓20%_, all rabbits received an initial fluid bolus of 30 mL/kg saline over 30 min. Additional fluid boluses, up to a cumulative total of 180 mL/kg over the 32-hour study period, were administered as needed to maintain MAP at pre-infection baseline levels (MAP_b_). If MAP_b_ could not be sustained with fluids alone, norepinephrine was administered and titrated from 0.05 to a maximum of 10 µg/kg/min. Lung-protective ventilation settings—including FiO₂ at 35%, flow rate of 4 L/min, respiratory rate of 35 breaths/min, peak inspiratory pressure (PIP) of 15 cmH₂O, positive end-expiratory pressure (PEEP) of 5 cmH₂O, and tidal volume of 6–7 mL/kg—were maintained throughout the study.

The primary endpoint was survival over the 32-hour treatment period. Animals were euthanized at 32 h or earlier if they met humane endpoints, defined as serum lactate >15 mmol/L and >50% reduction in MAP from baseline, along with evidence of respiratory failure or terminal shock. Survival was 75% (9/12) in the VIP plus ciprofloxacin group compared to 0% (0/12) in the VIP plus placebo group (*P* < 0.001 by log-rank test; Fig. 5C). Rabbits in the VIP plus placebo group exhibited high bacterial burdens, while those receiving VIP plus ciprofloxacin had CFU counts in target organs below the detection limit (log₁₀ CFU = 1.72) (Fig. 5D).

The cumulative amount of saline used for fluid challenge (median, IQR) was 98 (92–114) mL/kg for rabbits in the VIP plus placebo group and 84 (62–127) mL/kg for rabbits in the VIP plus ciprofloxacin group (*P* = 0.18 by Mann–Whitney U test). The cumulative amount of norepinephrine used for fluid challenge was 2.1 (1.8–3.2) µg/kg/min for rabbits in the VIP plus placebo group and 1.2 (0.7-3–1) µg/kg/min for rabbits in the VIP plus ciprofloxacin group (*P* = 0.20 by Mann–Whitney U test).

### Combined ciprofloxacin and VIP supportive care stabilized hemodynamics and mitigated myocardial injury

Animals receiving VIP + ciprofloxacin maintained higher mean arterial pressures (MAP) throughout the study, with terminal MAP nearly doubling that of VIP + placebo animals (50 mm Hg [41–57] vs 28 mm Hg [25–32]; *P* < 0.001) (Table 1). To evaluate the physiological effects of combining antimicrobial therapy with intensive care–style supportive management, we performed longitudinal analyses of arterial blood gases, clinical chemistry, and hematologic parameters at four predefined time points: pre-infection baseline, treatment initiation (TTx), 6 h post-treatment (TTx + 6 h), and the terminal endpoint (Table 1). Progressive metabolic acidosis observed in placebo-treated rabbits was attenuated by ciprofloxacin therapy, as indicated by higher arterial pH (7.30 [7.11–7.40] vs 6.84 [6.68–6.92]; *P* = 0.003), improved base excess (−7.9 [−17.7 to −2.7] vs −26.9 [−30.5 to −25.3]; *P* = 0.002), and lower serum lactate (6.3 [2.9–9.8] vs 14.1 [11.4–16.0]; *P* = 0.005) at terminal endpoint. Markers of cardiac injury were markedly reduced at TE in the VIP + ciprofloxacin group compared with the VIP + placebo group: CK-MB levels were 15.8 (4.7–30.8) ng/mL vs 147.3 (107.2–254.5) ng/mL (*P* = 0.001), and myoglobin levels were 66 (4–626) ng/mL vs 4575 (2,6786,900) ng/mL (*P* < 0.001), although cardiac troponin I values were not significantly different between groups (Table 1).

Profound leukopenia and thrombocytopenia observed at treatment initiation (TTx) recovered substantially in ciprofloxacin-treated rabbits (Table 1). By terminal endpoint, total white blood cell counts were significantly higher in the VIP plus ciprofloxacin group (8.74 [5.14–10.35] × 10³/µL) than in placebo-treated controls (2.8 [1.81–3.95] × 10³/µL; *P* < 0.001), accompanied by restoration of neutrophil (2.03 [1.04–5.52] vs 0.27 [0.14–0.52] × 10³/µL; *P* < 0.001) and monocyte (0.74 [0.35–1.37] vs 0.05 [0.03–0.11] × 10³/µL; *P* < 0.001) subsets. Platelet counts also increased significantly (136 [86–203] vs 68 [52–86] × 10³/µL; *P* = 0.005), consistent with recovery from consumptive coagulopathy.

## DISCUSSION

The development of effective therapies for *P. aeruginosa* sepsis remains a major clinical challenge, particularly in the context of rising multidrug resistance and high mortality rates associated with septic shock (1). Here, we developed and validated a rabbit model of *P. aeruginosa*-induced septic shock that closely recapitulates key clinical hallmarks of human disease, including the biphasic hemodynamic profile and terminal cardiovascular collapse. Importantly, the model incorporates ICU-style monitoring and protocolized therapeutic interventions, providing a clinically relevant platform for evaluating both antimicrobial efficacy and supportive care strategies (14, 21).

Our natural history study in the rabbit model of *P. aeruginosa*-induced septic shock showed a rapid hyperdynamic response followed by a reproducible transition to hypodynamic shock, marked by profound hypotension, rising central venous pressure, and decreased cardiac output (Fig. 1). These changes were accompanied by metabolic acidosis, cytopenias, and increased levels of acute myocardial injury biomarkers—hallmarks of clinical features of human septic shock (Fig. 2–3 and Fig. S3) (17, 18).

To facilitate timely and clinically actionable interventions, we identified T_MAP↓20%_, corresponding to a 20% decline in MAP from pre-infection base, as a practical physiologic marker of impending cardiovascular decompensation. This model-derived threshold consistently preceded terminal endpoints by several hours, thereby defining a reproducible window during which therapeutic interventions could be initiated (Fig. 1). Using this physiologic marker, we demonstrated that ciprofloxacin monotherapy—administered at a human-equivalent dosing regimen of 30 mg/kg q8h via 1-hour infusion (Fig. 4)—modestly prolonged survival and reduced bacterial burden but failed to prevent lethal septic shock in most animals (Fig. 5A and B). These findings align with clinical observations that antibiotic monotherapy is often insufficient in the absence of hemodynamic stabilization (22, 23). Incorporation of VIP supportive care, including mechanical ventilation, fluid resuscitation, and titratable vasopressor administration, significantly improved outcomes when combined with ciprofloxacin (Table 1; Fig. 5C and D). While ciprofloxacin therapy resulted in bacterial eradication, the addition of protocolized VIP supportive care was essential for maintaining hemodynamic stability, preventing progression to cardiovascular collapse, and rescuing 75% of animals from otherwise fatal septic shock.

Our development of the rabbit model of *P. aeruginosa* hyperdynamic septic shock aligns with the core principles set forth by the Minimum Quality Threshold in Pre-Clinical Sepsis Studies (MQTiPSS) initiative, an international expert consensus effort aimed at improving the design, reproducibility, and translational value of animal sepsis models (5). MQTiPSS emphasized the importance of using clinically relevant pathogens, defining physiological criteria for sepsis diagnosis, incorporating ICU-standard monitoring and supportive care, and establishing clinically relevant therapeutic windows for intervention (5, 21, 24, 25). Notably, MQTiPSS reported that over 70% of highly cited preclinical sepsis studies published between 2003 and 2017 omitted fluid resuscitation entirely and that only 3% of the remaining studies titrated fluids to a defined physiological or organ-support target (18, 21). By addressing these critical gaps, including advanced hemodynamic monitoring, predefined MAP-based intervention triggers, VIP supportive care, and antimicrobial dosing guided by human PK/PD targets, our rabbit model fulfills multiple MQTiPSS-recommended criteria (5, 21, 24, 25). These features position the rabbit model as a viable platform for preclinical testing of candidate therapeutics under conditions that closely mimic human sepsis care.

Findings from our rabbit model are supported by earlier work in a canine model of *Escherichia coli* septic shock, which demonstrated that combined antibiotic therapy and cardiovascular support with fluids and dopamine significantly improved survival, whereas either therapy alone was largely ineffective (26). Moreover, incorporation of ventilation, fluid resuscitation, and vasopressor support into preclinical sepsis models is widely regarded as critical for translational relevance (5, 27). Consistent with these observations, ciprofloxacin monotherapy in our model was insufficient to prevent septic shock and death in most rabbits, whereas the addition of protocolized VIP supportive care rescued 75% of animals (Fig. 5). Together, these data underscore the importance of combining targeted antimicrobial therapy with hemodynamic stabilization in septic shock.

Rodent models have traditionally served as the backbone of preclinical sepsis research due to their affordability and scalability for high-throughput screening (6, 28). Moreover, mice are highly resistant to LPS and often fail to exhibit the hyperdynamic-to-hypodynamic transition seen in clinical septic shock (9, 11). In contrast, rabbits exhibit susceptibility to LPS (10, 11), making them well-suited for modeling human septic shock. In addition to these pathophysiologic advantages, rabbits permit reliable intravenous drug administration using computer-controlled syringe pumps, serial blood sampling, and within-animal pharmacokinetic profiling, thereby facilitating PK/PD-guided dosing strategies that are difficult to implement in rodent models (Fig. 4) (29).

Our rabbit model provides real-time access to arterial and venous pressures and thermodilution-based cardiac output, enabling hemodynamic monitoring of shock dynamics. Importantly, this model supports protocolized VIP interventions, including mechanical ventilation, titratable fluid resuscitation, and vasopressor administration—elements largely absent from rodent sepsis studies but essential to human critical care (5, 21, 24, 25). Thus, the rabbit model may help bridge the gap between rodent preclinical efficacy testing of drug candidates and clinical trial outcomes.

Collectively, these results establish the rabbit model as a robust and clinically relevant platform for testing the efficacy of antimicrobial agents and supportive strategies in severe *P. aeruginosa* sepsis. The model enables precise timing of therapeutic intervention, facilitates PK/PD-guided dosing, and provides a rigorous framework for evaluating new drug candidates in a setting that mirrors human critical care. Future studies will expand upon this work by incorporating multidrug-resistant clinical isolates, evaluating novel antimicrobial combinations, and refining supportive care protocols to further align with evolving standards in sepsis management.

## MATERIALS AND METHODS

### Study design

To develop a clinically relevant rabbit model of *P. aeruginosa*-induced hyperdynamic septic shock, we conducted a two-part study incorporating advanced ICU-style monitoring and therapeutic interventions reflective of human clinical care. For the natural history study, seven mechanically ventilated rabbits were intravenously challenged with a lethal dose of *P. aeruginosa* strain 6206 to characterize disease progression and establish a reproducible physiological trigger for intervention. Mean arterial pressure (MAP), cardiac output, central venous pressure, and other hemodynamic and biochemical markers were monitored to identify the transition from hyperdynamic to hypodynamic shock. The time when MAP declined at least 20% from baseline (T_MAP↓20%_) was identified as a clinically actionable threshold indicating cardiovascular decompensation. For the therapeutic efficacy studies, 12 rabbits were randomized at T_MAP↓20%_ to receive either ciprofloxacin monotherapy (30 mg/kg, every 8 h × 3 doses) or placebo (saline).

To evaluate whether life-sustaining supportive care enhances antimicrobial efficacy, an additional 24 infected rabbits were randomized at T_MAP↓20%_ to receive ciprofloxacin or placebo in conjunction with protocolized supportive care modeled after the clinical “VIP” strategy: **V**entilation, **I**nfusion, and **P**ressors. All animals were maintained under low tidal volume ventilation. At T_MAP↓20%_, animals received an initial 30 mL/kg bolus of normal saline over 30 minutes, with additional boluses administered to maintain MAP at baseline levels (MAP_b_), up to a maximum of 180 mL/kg. If fluid resuscitation failed to restore MAP_b_, norepinephrine was titrated from 0.05 to 10 µg/kg/min. This design allowed assessment of antimicrobial efficacy under standard ICU interventions, enabling rigorous evaluation of both monotherapy and combined therapy outcomes.

Pa6206, originally isolated from a human corneal infection (30), was selected for rabbit model development due to its increased virulence compared to other laboratory strains (31–33). Pa6206 is susceptible to all antipseudomonal agents, including ciprofloxacin (MIC, 0.5 µg/mL).

See Supplemental material for detailed information on animal history, anesthesia, ventilation and instrumentation, bacterial strain and inoculum preparation, pharmacokinetics and AUC/MIC calculation, necropsy and microbial burden analysis, and statistical analysis.

## SUPPLEMENTAL MATERIAL

Supplemental Methods and Figures aac.01936-25-s0001.docx Fig. S1 to S3.

ASM does not own the copyrights to Supplemental Material that may be linked to, or accessed through, an article. The authors have granted ASM a non-exclusive, world-wide license to publish the Supplemental Material files. Please contact the corresponding author directly for reuse.
