# Supplementary material for: Development and validation of a rabbit model of Pseudomonas aeruginosa hyperdynamic septic shock for preclinical drug development
Source: Antimicrob Agents Chemother. 2026 Jun 15;70(8):e01936-25. doi: 10.1128/aac.01936-25 (PMC13435728; doi:10.1128/aac.01936-25)
Supplement: Supplementary file 1 [file aac.01936-25-s0001.docx]

**SUPPLEMENTAL METHODS**

**Animals.** Female New Zealand White rabbits (3.0–3.4 kg; Western Oregon Rabbit Co.) were housed under standard conditions with ad libitum access to food and water. Animals were acclimated for at least 48 h before experimentation, and only healthy rabbits without diarrhea were used.

**Anesthesia, Ventilation, and Instrumentation.** Rabbits were sedated with subcutaneous buprenorphine (0.01–0.05 mg/kg), followed 30 minutes later by intramuscular injection of ketamine (36 mg/kg) and xylazine (5.2 mg/kg). Once adequate sedation was achieved, animals were intubated using a 3.0 mm cuffed endotracheal tube and mechanically ventilated with the Engler ADS 2000 ventilator. A lung-protective ventilation strategy was employed (1), consisting of a peak inspiratory pressure (PIP) of 15 centimeters of water (cm H₂O), tidal volume of 5–7 milliliters per kilogram (mL/kg), positive end-expiratory pressure (PEEP) of 5 cm H₂O, respiratory rate of 30 breaths per min, fraction of inspired oxygen (FiO₂) at 35%, flow rate of 4 liters per minute (L/min), and inhaled isoflurane (2.0–2.5%) for anesthesia maintenance. Ventilator settings were held constant throughout the study.

Each rabbit was surgically instrumented for hemodynamic monitoring (2). The right carotid artery was cannulated with either a Pulse Contour Cardiac Output PiCCO thermodilution catheter (Natural History Study) or an 18-gauge intravenous catheter (Efficacy Studies). The right internal jugular vein was catheterized using a 4-French (4F) dual-lumen catheter to allow for fluid and drug administration. A continuous infusion of Normosol-R solution with 5% dextrose was maintained at 2.5 mL/kg/hour. All arterial and venous catheters were flushed continuously with saline containing heparin at a concentration of 1 unit per mL (U/mL).

Physiological monitoring was conducted using a Philips MP90 system to record mean arterial pressure (MAP), central venous pressure (CVP), electrocardiogram (ECG), rectal temperature, oxygen saturation (SpO₂), and end-tidal carbon dioxide (ETCO₂). Cardiac output was measured every 2 or 4 h using thermodilution.

**Bacterial Strain and Inoculum Preparation.** Pa6206 was streaked from -80°C frozen stock onto a 5% sheep blood agar plate (Remel) and incubated overnight at 37°C. Five well-isolated colonies were streaked into a lawn on a blood agar plate and incubated for 10 h at 37°C. Cells were harvested, washed in lactated Ringer’s solution (LRS), and resuspended to an OD₆₀₀ of 1.30, corresponding to 3 x 10^9^ CFU/2.2 mL. Inocula were injected via a marginal ear vein over 1 minute. Inoculum CFU concentrations were confirmed by serial dilution and plating onto blood agar plates.

**Natural History Study.** For the natural history study, rabbits were challenged intravenously with 3 x 10^9^ CFU Pa6206 and monitored continuously. Terminal organ bacterial burdens were quantified by CFU plating.

**Efficacy Studies.** For two efficacy studies, rabbits were challenged intravenously with 3 x 10^9^ CFU *P. aeruginosa* strain 6206. In the first efficacy study, rabbits were randomized at the time of a 20% MAP decline (T_MAP↓20%_) to receive either ciprofloxacin (30 mg/kg q8h × 3 doses) or placebo (saline). In the second efficacy study, rabbits were randomized to receive ICU-style supportive care (fluid resuscitation + norepinephrine) plus either ciprofloxacin or placebo. Supportive care began at T_MAP↓20%_ with a 30 mL/kg saline bolus over 30 min, repeated up to 180 mL/kg as needed. If MAP could not be maintained, norepinephrine (0.05–10 µg/kg/min) was titrated to restore baseline MAP. Ciprofloxacin (2 mg/ml IV solution in 5% dextrose, Hospira) was infused over 1 hour via syringe pump. Animals were monitored up to 32 h post-treatment or euthanized earlier based on humane endpoints (lactate >15 mmol/L, MAP reduction >50%).

**Pharmacokinetics and AUC/MIC Calculation.** Serial blood samples were collected at predefined time points (0, 1.08, 1.5, 2.0, 2.5, 4.0, 6.0, and 8.0 h) following ciprofloxacin administration for pharmacokinetic analysis. Total ciprofloxacin plasma concentrations (bound and unbound) were determined in infected and uninfected rabbits following intravenous ciprofloxacin dosing using liquid chromatography tandem spectrometry (LC-MS/MS). The lower limit of quantification was 0.08 µg/ml for ciprofloxacin in rabbit plasma. Serial blood samples were collected after each dose for PK analysis, and AUC0-24h/MIC ratios were computed based on total drug exposure using noncompartmental analysis implemented in PKSolver v2.0 (3).

**Necropsy and Microbial Burden Analysis.** At necropsy, organs were weighed and sampled for microbiological analysis. Lung, heart, liver, spleen, and kidneys were homogenized, serially diluted, and plated on 5% sheep blood agar plates (Remel) for CFU enumeration.

**Statistical Analysis.** Survival curves were analyzed by log-rank test using GraphPad Prism v9 (College Station, TX). Nonparametric comparisons between experimental groups (VIP + placebo vs. VIP + ciprofloxacin) at four time points (pre-infection, TTx, TTx + 6 h, and TE) were performed using the Mann–Whitney test, with *P*-values adjusted using the Holm–Šidák method to control the family-wise error rate across multiple comparisons.

**REFERENCES**

1.	**Nguyen NTQ, Gras E, Tran ND, Nguyen NNY, Lam HTH, Weiss WJ, Doan TNM, Diep BA.** 2021. Pseudomonas aeruginosa Ventilator-Associated Pneumonia Rabbit Model for Preclinical Drug Development. Antimicrob Agents Chemother **65:**e0272420.

2.	**Nguyen NTQ, Doan TNM, Sato K, Tkaczyk C, Sellman BR, Diep BA.** 2023. Monoclonal antibodies neutralizing alpha-hemolysin, bicomponent leukocidins, and clumping factor A protected against Staphylococcus aureus-induced acute circulatory failure in a mechanically ventilated rabbit model of hyperdynamic septic shock. Front Immunol **14:**1260627.

3.	**Zhang Y, Huo M, Zhou J, Xie S.** 2010. PKSolver: An add-in program for pharmacokinetic and pharmacodynamic data analysis in Microsoft Excel. Comput Methods Programs Biomed **99:**306-314.

**Supplemental Figure 1.** Natural history of hemodynamics during P. aeruginosa septic shock in mechanically ventilated rabbits. Seven anesthetized New Zealand White rabbits (A–G) were challenged intravenously at 0 h with 3 x 10^9^ CFU of P. aeruginosa strain 6206 and monitored continuously without therapy. Each panel shows the time course of a single hemodynamic parameter across all animals: (A) heart rate (HR), (B) mean arterial pressure (MAP), (C) central venous pressure (CVP), (D) cardiac output (CO), (E) systemic vascular resistance (SVR), and (F) stroke volume (SV). Traces end at the humane/terminal endpoint (TE) for each rabbit. Colors correspond to individual animals as indicated in the legend. These same data are also presented in Figure 1, where all parameters are shown for each individual animal to allow visualization of the temporal coordination of physiological changes within animals.

**Supplemental Figure 2. Natural history of *P. aeruginosa* septic shock in rabbits.** (**A**) Kaplan–Meier survival curve for rabbits (n=7) intravenously challenged with *P. aeruginosa* strain 6206 at a lethal dose of 3×10⁹ CFU. All animals succumbed to infection within 48 hours (median survival, 16.5 h). (**B**) Quantification of bacterial burdens at necropsy showing disseminated infection across major organs. Each symbol represents an individual animal; bars denote median ± interquartile range.

**Supplemental Figure 3. Biomarkers of organ injury during *P. aeruginosa* septic shock in the rabbit natural history study.** Blood samples at baseline (0 h), 4 h post-infection, the time of a 20% decline in mean arterial pressure (MAP↓20%), and the terminal endpoint (TE) were analyzed for biomarkers of acute organ injury. Panels show concentrations of (**A**) cardiac troponin I (cTnI), (**B**) creatine kinase–MB (CK-MB), and (**C**) myoglobin as indicators of myocardial injury; (**D**) aspartate aminotransferase (AST), (**E**) alanine aminotransferase (ALT), and (**F**) AST/ALT ratio as markers of hepatocellular injury; (**G**) total bilirubin, (**H**) blood urea nitrogen (BUN), and (**I**) creatinine as indicators of renal dysfunction; and (**J**) total protein and (**K**) albumin as measures of liver function. Each colored trace represents one of the same seven rabbits depicted in Figure 1.
